# High-Energy Mechanical Milling-Driven Reamorphization in Glassy Arsenic Monoselenide: On the Path of Tailoring Special Molecular-Network Glasses

**DOI:** 10.3390/ma14164478

**Published:** 2021-08-10

**Authors:** Yaroslav Shpotyuk, Pavlo Demchenko, Oleh Shpotyuk, Valentina Balitska, Catherine Boussard-Pledel, Bruno Bureau, Zdenka Lukáčová Bujňáková, Peter Baláž

**Affiliations:** 1 Institute of Physics, University of Rzeszow, 1, Pigonia Str., 35-959 Rzeszow, Poland; 2 Ivan Franko National University of Lviv, 1, Universytetska Str., 79000 Lviv, Ukraine; pavlo.demchenko@lnu.edu.ua; 3 Faculty of Science and Technology, Jan Dlugosz University in Czestochowa, 13/15, al. Armii Krajowej, 42-200 Czestochowa, Poland; o.shpotyuk@ujd.edu.pl; 4 Department of Optical Glass and Ceramics, Vlokh Institute of Physical Optics, 23, Dragomanov Str., 79005 Lviv, Ukraine; 5 Department of Physics and Chemistry of Combustion, Lviv State University of Life Safety, 35, Kleparivska Str., 79007 Lviv, Ukraine; vbalitska@yahoo.com; 6 Univ Rennes, CNRS, ISCR (Institut des Sciences Chimiques de Rennes), UMR 6226, F-35000 Rennes, France; catherine.boussard@univ-rennes1.fr (C.B.-P.); bruno.bureau@univ-rennes1.fr (B.B.); 7 Institute of Geotechnics of Slovak Academy of Sciences, 45, Watsonova Str., 04001 Košice, Slovakia; bujnakova@saske.sk (Z.L.B.); balaz@saske.sk (P.B.)

**Keywords:** high-energy mechanical milling, medium-range structure, intermediate-range ordering, extended-range ordering, arsenic monoselenide, X-ray powder diffraction

## Abstract

The impact of high-energy milling on glassy arsenic monoselenide g-AsSe is studied with X-ray diffraction applied to diffuse peak-halos proper to intermediate- and extended-range ordering revealed in first and second sharp diffraction peaks (FSDP and SSDP). A straightforward interpretation of this effect is developed within the modified microcrystalline approach, treating “amorphous” halos as a superposition of the broadened Bragg diffraction reflexes from remnants of some inter-planar correlations, supplemented by the Ehrenfest diffraction reflexes from most prominent inter-molecular and inter-atomic correlations belonging to these quasi-crystalline remnants. Under nanomilling, the cage-like As_4_Se_4_ molecules are merely destroyed in g-AsSe, facilitating a more polymerized chain-like network. The effect of nanomilling-driven molecular-to-network reamorphization results in a fragmentation impact on the correlation length of FSDP-responsible entities (due to an increase in the FSDP width and position). A breakdown in intermediate-range ordering is accompanied by changes in extended-range ordering due to the high-angular shift and broadening of the SSDP. A breakdown in the intermediate-range order is revealed in the destruction of most distant inter-atomic correlations, which belong to remnants of some quasi-crystalline planes, whereas the longer correlations dominate in the extended-range order. The microstructure scenarios of milling-driven reamorphization originated from the As_4_Se_4_ molecule, and its network derivatives are identified with an ab initio quantum-chemical cluster modeling code (CINCA).

## 1. Introduction

In the past decades, nanoscopic substances functionalized through different nanostructurization routines have attracted widespread attention in the materials science community in view of their unique and very important applications [1,2]. This is why the technology of high-energy mechanical milling (MM), sometimes also termed as nanomilling due to the unprecedented macro-to-nanoscopic transferring ability tending material systems towards an out-of-equilibrium high-entropy state, represents the most promising challenge in contemporary materials engineering [3,4,5,6]. Typically, the parent substances subjected to nanostructurization under MM are crystalline ones, since the consequences of nanomilling are hidden for amorphous solids prepared by rapid freezing from a melt (the melt-quenching, MQ). Being subjected to a destructive influence generating a huge number of structural defects, disordered solids show depressed glass-transition temperatures *T_g_* and accelerated physical ageing [7], while the realistic origin of responsible structural transformations has remained rather unclear.

A great plethora of amorphous materials such as glassy arsenoselenides g-As-Se possess principally different conformations associated with molecular and network entities [8,9,10,11,12], their functionalization being defined by system escape towards preferential atomic arrangement. It was found in a group of As-rich over-stoichiometric thioarsenides g-As_x_Se_100−x_ (χ > 40) that molecular-to-network transition could be initiated mechanochemically by treatment in a high-energy ball mill [13], allowing tailoring of advanced amorphous substances with guided properties.

Current research is aimed at studying nanomilling-driven molecular-to-network amorphization transition in glassy arsenic monoselenide g-AsSe (viz. As_50_Se_50_ or tetra-arsenic tetraselenide As_4_Se_4_), which is representative of a canonical over-stoichiometric As-Se glassy system possessing mixed molecular-network structural conformations [8,9,10,11,12]. With this in mind, the most plausible *reamorphization* scenarios in the molecular prototype of this compound (As_4_Se_4_) will be examined by employing X-ray powder diffraction (XRPD) analysis supplemented with a materials-computational approach based on ab initio quantum-chemical modeling within CINCA (cation-interlinked network cluster approach) [14,15].

## 2. Materials and Methods

### 2.1. Sample Preparation Routines

The glassy arsenic monoselenide g-AsSe was prepared by the conventional MQ route [10,11,12,13]. The melt-rocking technological operation [16] was employed to prepare the homogeneous g-AsSe specimens. The sealed ampoule with 99.999% pure elemental As and Se taken in the stoichiometric ratio was heated in a rocking furnace up to 650 °C for 6 h and homogenized for 10 h. At the finishing stage, the ampoule was placed vertically, cooled down to 500 °C (in 1 h), and finally quenched in water. To eliminate mechanical strains that appeared after rapid cooling, glass was annealed at 120 °C for 1 h. The ingot extracted from the ampoule was completely amorphous, as it followed from XRPD analysis, conch-like fracture, and IR transparency of obtained samples.

The macroscopic room-temperature density of this MQ-derived alloy *ρ* = 4.490 g⋅cm^−3^ (measured in ethanol by the Archimedes displacement method) and glass-transition temperature *T_g_* = 166 °C (the mid-onset point on the differential scanning calorimetry curve obtained with 10 °C/min heating rate using TA Instrument Q20 calorimeter) show good correlation with known glassy counterpart g-AsSe [8,9,10,11,12]. The mean inter-atomic spacing *d_s_^m^* = 3.79 Å calculated from atomic density *ρ* was close to the maximum among over-stoichiometric arsenoselenides g-As_x_Se_100−x_ (χ > 40) [13].

The nanomilling route [3,4,5,6] was employed to transfer MQ-derived g-AsSe into nanoscopic fine-grained state. Preliminarily, the coarse-grained pieces of g-AsSe were subjected to rough powdering followed by sieving under 200 μm. Then, the MM activation of the prepared material (~3 g) was performed using Pulverisette 6 (Fritsch, Idar-Oberstein, Germany) planetary ball mill operated in a dry mode at Ar atmosphere. The nanomilling was performed in 250 mL WC chamber (loaded with 50 WC balls, each having 10 mm in diameter). The rotational speed of 500 min^−1^ was employed for 60 min. Under such conditions, the energy transferring to fine-powdered g-AsSe estimated over specific grinding work in the Pulverisette 6 mill [17,18,19] was estimated to approach ~320 kJ/g. The high-energy MM under these conditions is known to ensure effective mechanochemical activation in a rich family of chalcogenide-type compounds [6,13,20,21,22,23,24,25,26,27,28,29,30].

### 2.2. Medium-Range Structure of Network Glass-Forming Substances by XRPD Analysis

The XRPD patterns were obtained in transmission mode using Cu *K*α_1_-radiation and curved Ge monochromator on the primary beam (STOE STADI P diffractometer) [31]. The measurements were performed with 0.015°2θ step and 0.480°2θ detector increment within a whole range. The scanning time was 500 s per step.

The amorphous phases in MQ- and MM-derived arsenic monoselenide g-AsSe were identified by XRPD with respect to diffuse “amorphous” peak-halos character for these disordered substances, particularly the FSDP (the first sharp diffraction peak), which is a signature of structural entities forming a so-called intermediate range ordering over a few tens of Å (reproduced in a reciprocal space near scattering vectors *Q*_1_ ≅ ~1–1.5 Ǻ^−1^) [32], and SSDP (the second sharp diffraction peak*,* in terms of [33]) or PDP (the principal diffraction peak*,* in terms of [34]), serving as signature of extended-range ordering (revealed in a reciprocal space near *Q*_2_~1.8–2.2 Ǻ^−1^). In glassy chalcogenides, the FSDP is typically observed as an extended peak-halo in the XRPD pattern at ~15–22°2θ, corresponding to real-space entities forming intermediate-range order of some network-forming structural motifs, while the SSDP is shifted to higher angles (~28–33°2θ) being ascribed to characteristic sizes of these motifs close to mean inter-atomic spacing *d_s_^m^* [32,33,34,35,36,37]. At higher angles of ~50–60°2θ (equivalent to *Q*_3_~3.3–4.0 Ǻ^−1^), the third extended peak-halo (not so sharp as FSDP or SSDP) known as TDP (the third diffraction peak) is observed in the XRPD patterns as a manifestation of the shortest nearest-neighbor interatomic separation in a glass [34].

Preliminary processing of XRPD patterns was performed using the data on arsenoselenide polymorphs close to AsSe taken from known databases [38,39], in part the JCPDS cards No. 65-2365 for monoclinic As_2_Se_3_, No. 71-0388 for monoclinic As_4_Se_4_, and No. 04-4979 for orthorhombic As_4_Se_3_. The crystallographic details of these phases were visualized employing the programs DIAMOND [40] and VESTA [41].

STOE WinXPOW 3.03 [42] and PowderCell 2.4 [43] software were employed to analyze the peak-halos on the XRPD patterns (normalized with respect to the maximum peak). The accuracy in the determination of position (2θ) and full width at half maximum (FWHM) of peak-halos was ±0.05°2θ. Furthermore, the scattering vector *Q* corresponding to the peak-halo and width Δ*Q* in a reciprocal space was calculated as *Q* = (4π/*λ*)⋅sinθ and Δ*Q* = (4π/*λ*)⋅sin(FWHM/2), respectively. The characteristic distance *R* served as spacing of peak-responsible quasi-periodicity and correlation length *L* over which this periodicity maintained were, respectively, determined as *R* = 2π/*Q* and *L* = 2π/Δ*Q* [30,31,32,33,34,35].

In addition, we treated the amorphous halos as arising from coordination spheres. The averaged inter-atomic distances *d_s_* between scattering centers defined as radii of the corresponding coordination spheres were calculated as in randomly packed multiparticulate systems [44,45,46,47]. In this case, the XRPD patterns are defined by the Ehrenfest relation 2*d_s_*⋅sinθ = 1.23⋅*λ* [48]. The error bar in the above linear parameters (*R*, *L*, and *d_s_*) does not exceed ± 0.1 Å.

### 2.3. Cluster Modeling of Molecular-Network Conformations in Covalent Substances

The configurations of geometrically optimized As_4_Se_4_ molecule and network-forming derivatives reconstructed by breaking this cage-like molecule on separate fragments linked with surroundings by means of Se_1/2_…Se_1/2_ bridges were simulated using ab initio quantum-chemical cluster-modeling code CINCA [14,15]. The HyperChem Release 7.5 software was used for modeling. The restricted Hartree–Fock self-consistent field method with split-valence double-zeta basis set and single polarization function 6-311G* was employed [49,50,51]. Geometrical optimization and single-point energy calculations were performed by the Fletcher–Reeves conjugate gradient method until reaching the root-mean-square gradient of 0.1 kcal/(Å·mol). The final cluster-forming energy *E_f_* was corrected on the energy of terminated H atoms transforming the network configuration to a molecular one according to procedure developed elsewhere [51,52] and recalculated with respect to the energy of single trigonal AsSe_3/2_ pyramid (*E_f_* = −72.309 kcal/mol) [15].

Thus, the CINCA cluster-modeling route allows adequate characterization of molecular and network clusters in covalent substances, parameterizing the most energetically favorable structure-transforming scenarios. To compare glass-forming ability of topological configurations accounting for small rings (typical for thioarsenides such as As_4_Se_n_, n = 3–6), the average number of Lagrangian constraints per atom *n_c_* was calculated for respective networks built of these clusters using the Phillips–Thorpe constraint-counting algorithm [53,54,55] with stretching and bending forces ascribed to intra-molecular covalent bonds within these clusters. Thus, within the developed approach, the most plausible scenarios of MM-driven structural transformations in the arsenical under consideration of defined composition were reconstructed in terms of potential energy landscape, showing the over-barrier transitions between the respective minima of cluster-forming energies *E_f_* corresponding to the atomic cluster conformations.

## 3. Results and Discussion

### 3.1. XRPD Parameterization in MQ-Derived g-AsSe

**Figure 1:**
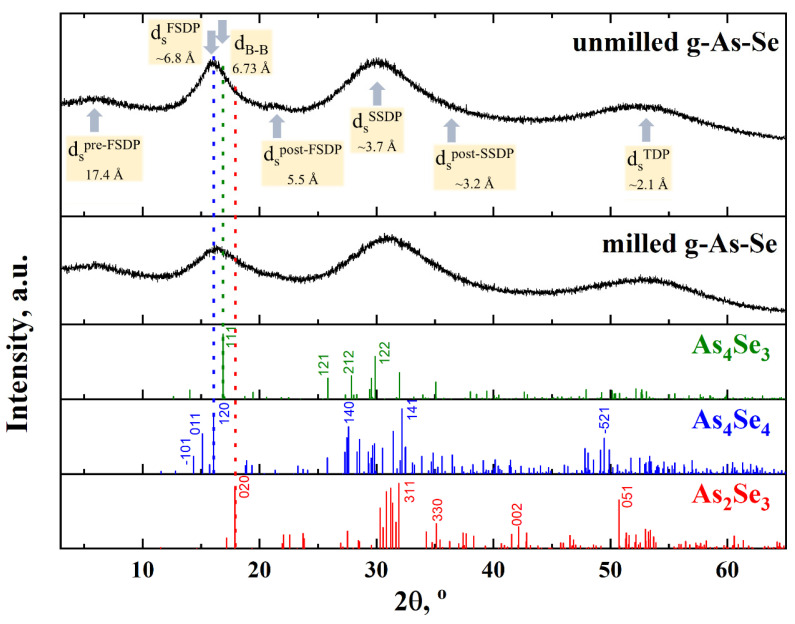
Experimental XRPD patterns of MQ-derived (unmilled) and MM-derived (milled) arsenic monoselenide g-AsSe demonstrating diffuse peak-halos ascribed to pre-FSDP (~6.2°2θ), FSDP (~16.0°2θ), post-FSDP (~20.0°2θ), SSDP (~29.8°2θ), post-SSDP (~33.9°2θ), and TDP (~52.6°2θ). The most prominent inter-atomic correlations *d_s_* corresponding to these features and centroid-centroid distance *d_B-B_* in the packing of As_4_Se_4_ cage-like molecules in the structure of monoclinic As_4_Se_4_ are indicated by arrows. Theoretical Bragg-diffraction reflexes of crystalline arsenic selenides of close chemical compositions such as As_4_Se_4_, As_4_Se_3_, and As_2_Se_3_ are given below for comparison (the XRPD patterns of g-AsSe are reconstructed from [13]).

The XRPD patterns collected from unmilled g-AsSe (Figure 1) demonstrate diffuse halos attributed to the FSDP, SSDP, and TDP at ~16.03°2θ, ~29.77°2θ, and ~52.64°2θ, respectively (see Table 1), corresponding to scattering vectors positioned at *Q^FSDP^*~1.138 Ǻ^−1^ (with width Δ*Q^FSDP^*~0.229 Ǻ^−1^), *Q^SSDP^*~2.096 Ǻ^−1^ (Δ*Q^SSDP^*~0.419 Ǻ^−1^), and *Q^TDP^*~3.617 Ǻ^−1^ (Δ*Q^TDP^*~0.624 Ǻ^−1^). As was shown in our preliminary research [13], a similar arrangement of peak-halos was also detected in the reduced X-ray structure factor derived from these XRPD profiles.

Within the microcrystalline approach applied to chalcogenide glass (distorted-layer/molecular model proposed by Vaipolin and Porai-Koshits [56] and developed by Gaskel [57] and Wright [58] for crystalline-like ordering in MQ-derived substances), the FSDP can be ascribed to quasi-periodicity in the distribution of some pseudo-planes for Bragg diffraction separating the succession of randomly packed cages. In the application to g-AsSe, this means a decisive role of remnants of distorted quasi-crystalline structures proper to compositionally close arsenic selenides such as As_4_Se_4_, As_3_Se_4_, and/or As_2_Se_3_ [13].

The experimental FSDP position in the MQ-derived g-AsSe (*R*~5.5 Å, see Table 1) is in excellent agreement with most intensive neighbouring Bragg diffraction lines arising from the (120) plane in the structure of molecular-type monoclinic As_4_Se_4_ at ~16.07°2θ corresponding to *inter-planar* distance *d* = 5.512 Å (*I* = 91.3%) [59]. Assuming equal contributions to the FSDP from most intensive neighbouring reflexes of all three structures, such as molecular-type As_4_Se_4_ with d(120) = 5.512 Å (*I* = 91.3%) [38,39,59], orthorhombic As_4_Se_3_ with d(111) = 5.243 Å (*I* = 100%) [59], and layer-type monoclinic As_2_Se_3_ with d (020) = 4.952 Å (*I* = 91.2%) [60,61,62,63], the FSDP-responsible characteristic distance *R* was estimated to be ~4.93 Å. If the FSDP is governed by equal contributions from molecular As_4_Se_4_ and layer As_2_Se_3_ crystalline polymorphs, this peak-halo occurs to be maximally shifted to higher scattering angles giving an equivalent characteristic distance *R* approaching ~4.77 Å. This misbalance testifies in favour of a preferential contribution to the FSDP from some remnants of a molecular-type As_4_Se_4_ structure in the glassy network of MQ-derived g-AsSe (see Figure 1).

The above consideration of most prominent inter-planar pseudo-crystalline correlations does not explain compositional variations in the FSDP in the binary As-Se system [13], other inputs to the FSDP being expected from inter-molecular correlations, i.e., some remnants of micromolecular structures. As was pointed out in [11], the structural features of over-stoichiometric glassy arsenic monoselenides such as g-AsSe could be imagined as a stacking of network entities based on Se-linked AsSe_3/2_ pyramids and molecular-type cage-like entities such as As_4_Se_4_, As_4_Se_3_, and/or As_4_. To demonstrate the spatial stacking of such cages, the geometrical barycentre of each molecule referred to as the “dummy atom” B was introduced in [62]. This simplification allows for estimating the shortest inter-molecular centroid-centroid distances in molecular structures (B-B), as well as their packing in some planes.

**Figure 2:**
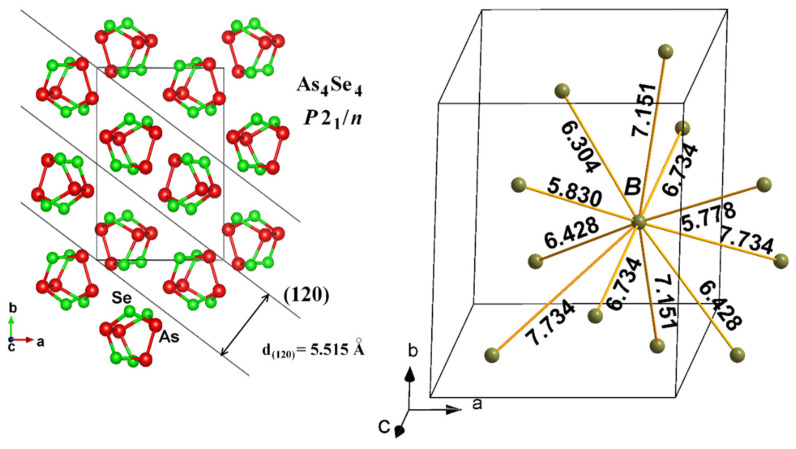
Fragment of crystalline structure of monoclinic As_4_Se_4_ reproduced at the basis of experimental data taken from [63] (the space group—*P*2_1_/*n*, the structure type—α-As_4_S_4_, realgar). On the left: packing of As_4_Se_4_ cage-like molecules forming a set of (120) crystallographic planes. On the right: all possible centroid-centroid distances *B*–*B* (in Å) between “dummy atoms” located at the barycentres of neighboring As_4_Se_4_ molecules (see [62] for details of “dummy atoms” identification).

Thus, the dense random packing of such molecules (viz., inter-molecular correlations) contributes to the XRPD patterns through the Ehrenfest-diffraction [48]. DIAMOND and VESTA software packages [40,41] were employed to visualize the fragment of the crystalline structure of monoclinic As_4_Se_4_ at the basis of data taken from [60] (see Figure 2). All possible inter-molecular centroid-centroid distances B-B and packing of As_4_Se_4_ cage-like molecules such as the *B*[*B*_11_] polyhedron together with the family of crystallographic planes corresponding to the (120) hkl reflection are presented in Figure 2. It is seen that each cage-like As_4_Se_4_ molecule is surrounded by 11 neighbors forming the *B*[*B*_11_] polyhedron, which is to be considered as incomplete distorted cubooctahedron in an fcc Cu-type structure with *d_B-B_* distances deviating from 5.778 Ǻ to 7.734 Ǻ. The averaged distance *d_B-B_*(As_4_Se_4_) approaches ~6.73 Ǻ. This distance accepted as the first coordination sphere in the dense-random packing of As_4_Se_4_ cages obeying the Ehrenfest equation remarkably corresponds to the FSDP positioned at *d_s_*~6.8 Å (see Table 1 and Figure 1).

Thus, we think that FSDP in the MQ-derived g-AsSe positioned at *Q^FSDP^*~1.138 Ǻ^−1^ corresponds to commensurable contributions from both inter-planar correlations (due to crystalline remnants of close compositions with the averaged Bragg diffraction distance *R* = 5.5 Ǻ) and inter-atomic correlations (due to third-order cation-cation pairs [13]) with the Ehrenfest diffraction distance *d_s_* = 6.8 Ǻ.

The non-elementary satellite nature of diffuse halos in g-AsSe is revealed through humps and asymmetric extensions evidencing a contribution from the Ehrenfest diffraction owing to most pronounced pair inter-atomic correlations. In the first place, this concerns post-FSDP, i.e., the shoulder at the high-angular side of the FSDP at ~20.00°2θ (see Figure 1). The ratio of the positions of both peaks (post-FSDP *Q^post-FSDP^* and FSDP *Q^FSDP^*) obey the simple relation *κ(FSDP)* = *Q^post-FSDP^*/*Q^FSDP^* = 1.24, approaching the Ehrenfest number (1.23) [48].

A similar phenomenon is found on the XRPD pattern in a vicinity of the SSDP at ~33.91°2θ (see Figure 1). An additional peak (reasonably referred to as the post-SSDP) located at the right-sided “tail” of the SSDP can be fitted to this diffuse peak-halo justifying its asymmetric shape. The relation *κ(SSDP)* = *Q^post-SSDP^*/*Q^SSDP^* = 1.13 (similar to that proper for post-FSDP and FSDP) is evidenced for this doublet as being close to the Ehrenfest number. Thus, we can assume that asymmetry in both peaks (FSDP and SSDP) is caused by the superposition of the broadened Bragg diffraction reflexes from some inter-planar correlations superimposed by the Ehrenfest diffraction reflexes from most prominent correlations between some atomic pairs belonging to these planes.

In contrast, the third diffraction peak-halo (the TDP) at ~(50–60)°2θ which is most probably associated with direct nearest-neighbor separation in a glass (due to *d_s_*~2.1 Ǻ, see Table 1) does not demonstrate a *doublet* structure.

The concept of the Ehrenfest diffraction appears to be the most suitable approach explaining another anomaly in the XRPD patterns of glassy chalcogenides known as pre-FSDP [64], which is revealed in the XRPD patterns as an additional peak-halo at low scattering angles ~(5–7)°2θ, i.e., in the diffraction angle region where there are no inter-planar reflexes from any possible crystalline counterparts. A diffuse peak-halo in this angular region (unreproducible in the structure factor [13]) can be explained as arising from *prolonged inter-atomic correlations* in a network approaching *d_s_*~15–20 Ǻ. The pre-FSDP did not show reproducible changes with a glass composition, such as under nanomilling (see Table 1). The similar invariant behavior is also character for the TDP positioned in the XRPD pattern of g-AsSe in the vicinity of scattering vector *Q^TDP^*~3.6 Å^−1^ (see Figure 1, Table 1).

### 3.2. XRPD Parameterization in MM-Derived g-AsSe

The nanomilling does not change the principal appearance of diffuse peak-halos on the XRPD profile of g-AsSe (see Figure 1), testifying that we are certainly dealing with a reamorphization process emerging as the MM-activated transition between two different amorphous states (amorphous-I-to-amorphous-II phase transition). The changes observed in the numerical parameters of diffuse peak-halos attributed to amorphous phases corresponding to MQ-derived (unmilled) and MM-derived (milled) specimens of arsenic monoselenide g-AsSe differ substantially (see Table 1).

After nanomilling, the FSDP shifts to higher angles of *Q^FSDP^*~16.42°2θ (corresponding to *Q^FSDP^*~1.165 Ǻ^−1^) and drastically grows in width on ~33% (see Table 1, Figure 1). Thereby, in milled g-AsSe, the spacing of FSDP-responsible periodicity *R* determined through *Q^FSDP^* decreases, and the correlation lengths *L* determined from Δ*Q^FSDP^* are reduced from 27.5 Ǻ to 20.7 Ǻ. Thus, MM results in a fragmentation impact on the correlation length *L* of quasi-periodic entities responsible for the FSDP.

The above MM-driven changes in the FSDP are concomitant with those detected in the SSDP, showing an increase in peak position *Q^SSDP^* and broadening in the width Δ*Q^SSDP^*. It is worth noting that nanomilling under current parameters does not influence the TDP in g-AsSe.

Thus, it seems reasonable to assume that structural entities responsible for both intermediate- and extended-range ordering in arsenic monoselenide g-AsSe are merely destroyed under high-energy MM, keeping the medium-range structure of the milled glass closer to layer-type g-As_2_Se_3_ [13,25].

The observed MM-driven changes in the FSDP are not strictly reproduced in the satellite high-angular post-FSDP (see Figure 1), linked with this peak-halo through the *κ(FSDP)* ratio of 1.26 (which is slightly above the Ehrenfest number, 1.23 [48]). Under high-energy MM, the most pronounced inter-atomic correlations responsible for post-FSDP became more depressed as compared with inter-planar correlations ascribed to the FSDP, thus ensuring the higher *κ* ratio in nanomilled g-AsSe. This means that a partial irreversible breakdown in intermediate-range ordering is expended in nanomilled g-AsSe over the destruction of most distant inter-atomic correlations belonging to remnants of quasi-crystalline planes contributing to the FSDP. As a result, asymmetry in the FSDP is reduced by MM.

With respect to post-SSDP, this satellite extension at the higher-angular “tail” of the SSDP linked with this peak-halo through *κ(SSDP)* = 1.13 is closer to the Ehrenfest number, thus meaning that longer inter-atomic distances (~3.2 Ǻ) become dominant in the extended-range ordering of nanomilled g-AsSe.

More specifically, the remnants of quasi-crystalline entities in MQ-derived g-AsSe responsible for inter-molecular correlations (with inter-centroid distances between As_4_Se_4_ cages *d_B-B_*(As_4_Se_4_) approaching ~6.73 Å), which contribute to the XRPD patterning through the Ehrenfest diffraction, are destroyed under nanomilling, since more depressed inter-planar quasi-crystalline correlations from these As_4_Se_4_ molecules (Figure 2) contribute to the XRPD patterns through the Bragg diffraction. Such a molecular-to-network reamorphization scenario shifts the broadened and more depressed FSDP towards the higher scattering vector *Q^FSDP^*~1.165, as it follows from the peak-halos positioned in arsenic monoselenide g-AsSe depicted on Figure 1.

The similar interrelation between FSDP and SSDP was observed in as-deposited arsenic triselenide films of 22 μm in thickness annealed below the glass transition temperature or illuminated by absorbed light [65]. Such changes were also detected by Sarsembinov et al. [66] in these 4–12 μm films prepared by thermal evaporation and ion-plasma sputtering.

To shed more light on these structural transformations, we shall refer readers to the amorphous-to-amorphous network transition observed in glassy arsenoselenides under growing pressure [67,68,69]. In the case of g-AsSe [67], the hydrostatic pressurization was shown to cause gradual densification, accompanied by the FSDP broadening (losses in intensity) and shifting to higher 2θ (a similar shift was also detected for other peaks, the SSDP and PDP). At ~16.6 GPa, the FSDP disappeared, signalizing a breakdown in the intermediate-range ordering in g-AsSe due to a transition from a quasi-layered to closely-packed network structure, this effect being reversible upon complete pressure release.

The high-energy MM (as in the current case) is an efficient way to stabilize such changes in the modified glasses as the result of nanomilling-driven irreversible reamorphization.

### 3.3. Cluster Modeling of Molecular-Network Reamorphization Scenarios in MM-Derived g-AsSe

Thus, molecular cage-like As_4_Se_4_ entities and their network-forming derivatives of the same composition play a governing role in the MM-activated reamorphization in arsenic monoselenide g-AsSe. The geometrically optimized configurations and stabilization cluster-forming energies *E_f_* for the cage-like As_4_Se_4_ molecule and atomic clusters reconstructed from this molecule by respective bond breaking at the positions of two-fold coordinated Se atoms polymerizing the molecular remainder in the covalent-bonded network though -Se- bridges have been identified by employing ab initio quantum-chemical simulation with CINCA modelling code [14,15].

**Figure 3:**
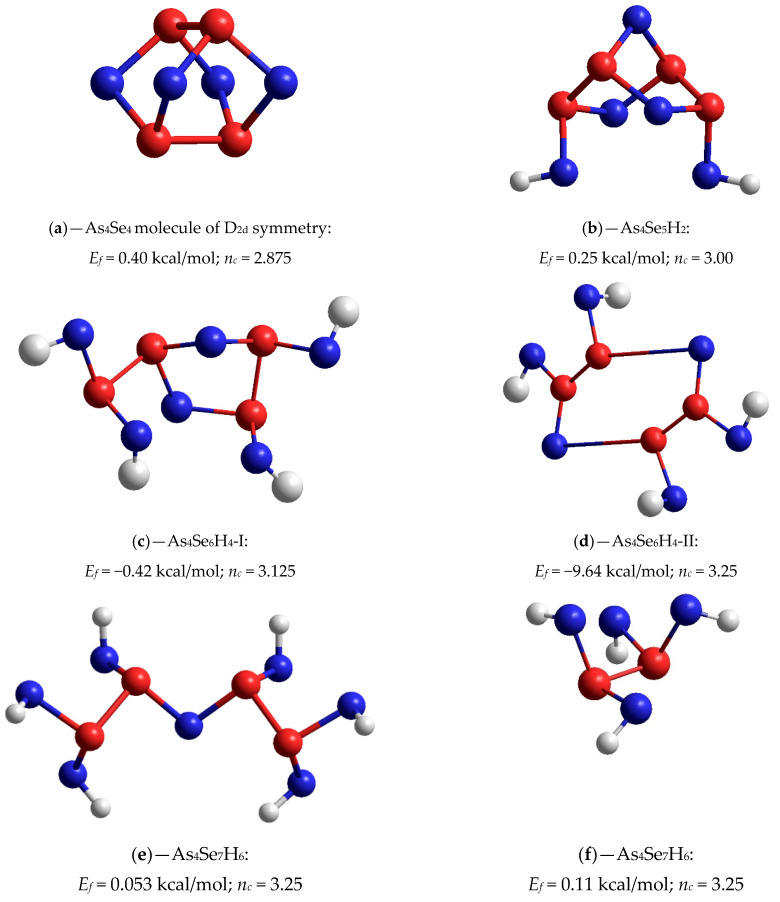
Optimized configurations of cage-like tetra-arsenic tetra-selenide molecule As_4_Se_4_ (**a**) and molecular prototypes of its network-forming derivatives reconstructed by single break in one of four Se positions (**b**, As_4_Se_5_H_2_), double break in two adjusted Se positions (**c**, As_4_Se_6_H_4_-I), double break in two opposite Se positions (**d**, As_4_Se_6_H_4_-II), triple break in three Se positions (**e**, As_4_Se_7_H_6_), and quadruple break in all Se positions (**f**, As_2_Se_4_H_4_). The cluster-forming energies *E_f_* are given with respect to the energy of single AsSe_3/2_ pyramid (having *E_f_* = −72.309 kcal/mol [15]). The terminated H atoms are grey-colored, Se and As atoms are blue- and red-colored, and bonds between atoms are denoted by respectively colored sticks.

The parent As_4_Se_4_ molecule of *D*_2*d*_ symmetry evolving a maximum number of small rings (four pentagons and four hexagons) built of eight As-Se bonds and two As-As bonds is depicted in Figure 3a. The cluster-forming energy for this cage-like molecule approaches *E_f_* = 0.40 kcal/mol (with respect to the energy of the AsSe_3/2_ pyramid, −72.309 kcal/mol [15]), which is the dominating value among all As_4_Se_4_-derived network-forming clusters shown in Figure 3.

If the whole glassy matrix is formed only from these As_4_Se_4_ cages, the number of topological constraints per atom *n_c_* = 2.875, which is smaller than the dimensionality of space (3.00), corresponding to an under-constrained (floppy) network. The geometrically optimized parameters of this molecule are in good agreement with intramolecular bond distances and angles refined from its crystalline counterpart, the monoclinic tetrameric arsenic selenide As_4_Se_4_ [59,70,71,72]. Thus, in this molecular cluster, the equivalent directly bonded As-Se distances occur to be very close to ~2.38 Å, As-As distances are compactly grouped near the average value of ~2.55 Å, and bond angles approach, respectively, 94.5° for ∠Se-As-Se, 97.5° for ∠As-Se-As, and 101.4° for ∠As-As-Se. The symmetry of the As_4_Se_4_ cage-like molecule is additionally confirmed by small deviations in the calculated values of the above bond distances and bond angles.

Among a group of network-forming derivatives reconstructed from this As_4_Se_4_ molecule, the most plausible is the network-forming cluster which can be imagined as appearing due to a single break in one of four Se atom positions, this being referred to as x1-As_4_Se_4_ (following the nomenclature in [26]). The optimized configuration of the molecular prototype of this cluster (As_4_Se_5_H_2_) is shown in Figure 3b. With respect to the calculated cluster-forming energy (*E_f_* = 0.25 kcal/mol), this cluster appears to be quite competitive with the parent As_4_Se_4_ molecule (in view of low barrier of molecular-to-network transition reaching Δ*E_f_* = 0.15 kcal/mol). Moreover, the network built of such atomic clusters (keeping only one hexagon and two pentagons as small rings shown on Figure 3b) is expected to possess a better glass-forming ability in view of their optimally constrained nature (*n_c_* = 3.00, which is with strict respect to space dimensionality).

Two network-forming clusters derived from the As_4_Se_4_ molecule through double breaking in the Se atom positions (x2-As_4_Se_4_-I and x2-As_4_Se_4_-II) are over-constrained, i.e., stress and rigid in view of *n_c_* > 3.00. The first of them with molecular prototype (As_4_Se_6_H_4_-I) shown in Figure 3c is reconstructed from this molecule due to breaking in two adjusted Se positions. This cluster keeping one pentagon-type ring in the atomic arrangement (thus resulting in *n_c_* = 3.125 for the whole network) is competitive with the former, since the cluster-forming energy *E_f_* reaches −0.42 kcal/mol (so that Δ*E_f_* = 0.67 kcal/mol). The second network cluster having *n_c_* = 3.25 with molecular prototype (As_4_Se_6_H_4_-II) shown in Figure 3d is derived from the As_4_Se_4_ molecule due to breaking in two opposite Se positions. This cluster suggests more energy gains to accommodate a hexagon-type ring with strongly deviated angular and linear parameters (some of the bond angles exceed ~120°, and bond lengths differ in the range of ~0.2–0.3 Å). Therefore, this network-forming cluster seems to be unfavorable because *E_f_* = −9.64 kcal/mol, resulting in unrealistically high Δ*E_f_* above ~10 kcal/mol with respect to the parent As_4_Se_4_ cage-like molecule.

With a further increase in the number of breaks in Se atom positions, the network-forming clusters derived from the cage-like As_4_Se_4_ molecule (the triple-broken x3-As_4_Se_4_ and quadruple-broken x4-As_4_Se_4_) attain a completely over-constrained chain-like network structure without any small rings with *n_c_* = 3.25 (see molecular prototypes of these clusters depicted in Figure 3e,f). The respective intrinsic angular and linear inter-atomic correlations in these x3-As_4_Se_4_ and x4-As_4_Se_4_ clusters slightly deviate from those proper to the parent As_4_Se_4_ molecule, resulting in a relatively small *E_f_* approaching 0.05 kcal/mol and 0.11 kcal/mol, respectively. The fully polymerized network amorphous structures can be built from these clusters, provided that the competitive portion of energy is gained under MM (Δ*E_f_* > 0.35 kcal/mol).

**Figure 4:**
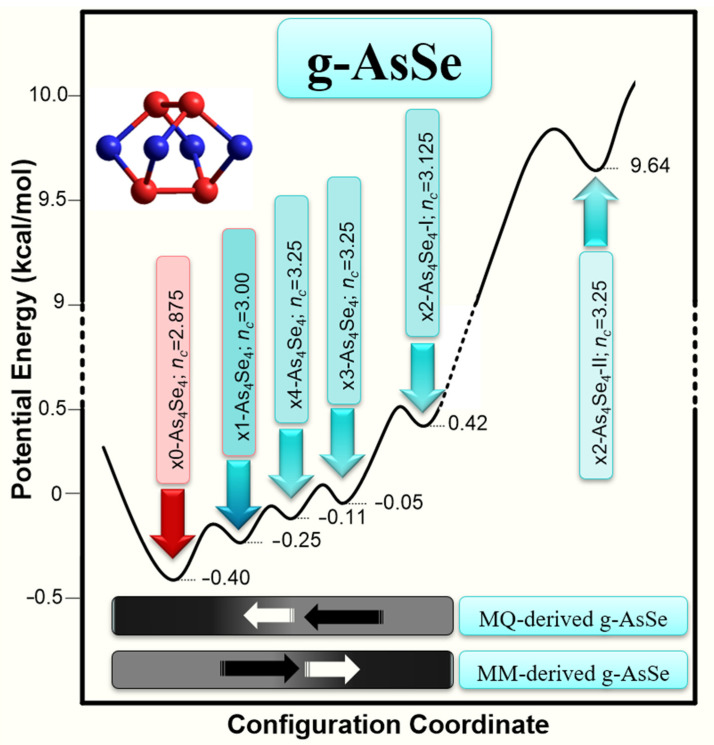
Potential energy landscape showing diversity of molecular-network cluster-related states in arsenic monoselenide g-AsSe. The cluster-forming energies *E_f_* (in kcal/mol) of As_4_Se_4_ cage-like molecule x0-As_4_Se_4_ and its network-forming derivatives are denoted below energy wells pointed out by respectively colored arrows (the atom-averaged number of topological constraints *n_c_* is given). The configuration coordinate domains corresponding to preferential stabilization of MQ-derived (unmilled) and MM-derived (milled) amorphous structures are black-shadowed below.

Thus, as it follows from the unified potential energy landscape in Figure 4 showing a diversity of mixed molecular-network states in arsenic monoselenide g-AsSe related to molecular As_4_Se_4_ entities, the resultant amorphous structure is stabilized under balance between x0-As_4_Se_4_ cage-like molecules (*E_f_* = 0.40 kcal/mol) and some As_4_Se_4_-related network-forming derivatives, these being as follows (in sequence of growing molecular-to-network transformation barrier, Δ*E_f_*): single-broken x1-As_4_Se_4_ (Δ*E_f_* = 0.15 kcal/mol), quadruple-broken x4-As_4_Se_4_ (Δ*E_f_* = 0.29 kcal/mol), triple-broken x3-As_4_Se_4_ (Δ*E_f_* = 0.35 kcal/mol), and double-broken x2-As_4_Se_4_-I (Δ*E_f_* = 0.82 kcal/mol).

In MQ-derived g-AsSe, the equilibrium is shifted towards glassy structures preferentially built of under-constrained molecules x0-As_4_Se_4_ (*n_c_* = 2.875) and optimally constrained network-forming clusters x1-As_4_Se_4_ (*n_c_* = 3.00), while the structure of MM-derived g-AsSe (nanomilled) is more defective, shifted towards over-constrained chain-like structural entities (such as x4-As_4_Se_4_ and x3-As_4_Se_4_ clusters having *n_c_* = 3.25 and even x2-As_4_Se_4_-I clusters with *n_c_* = 3.125). It worth mentioning that there is no notable left barrier for x4-As_4_Se_4_ clusters because of the polymerization trend tending to cause them to be incorporated in a network. Because of the low barrier between states of the x0-As_4_Se_4_ and x1-As_4_Se_4_ clusters (Δ*E_f_* = 0.15 kcal/mol, see Figure 4), the stabilized structure of MQ-derived arsenic monoselenide g-AsSe, albeit being mainly built of molecular entities, is not completely molecular in a strict crystallographic manner (i.e., possessing a monoclinic structure built of As_4_Se_4_ cage-like molecules as depicted on Figure 2, isomorphous with the realgar structure [63,70,71,72]). The enhanced intermediate- and extended-range ordering revealed in the XRPD patterns of MQ-derived (unmilled) g-AsSe preferentially results from these molecular entities (x0-As_4_Se_4_) and optimally constrained network clusters (x1-As_4_Se_4_), frozen under rapid cooling from a melt (see Figure 4).

Under more non-equilibrium conditions of nanomilling producing a huge number of structural defects due to mechanical grinding [17,18,19], the contribution of network-forming entities in the structure of arsenic monoselenide g-AsSe is evidently enhanced, preferentially due to the generation of chain-like x4-As_4_Se_4_ and x3-As_4_Se_4_ clusters. The obviously depressed molecularity in such disordered materials means nanomilling-driven molecular-to-network reamorphization, essentially modifying the arrangement of diffuse peak-halos in their XRPD patterning (see Figure 1).

## 4. Conclusions

The impact of high-energy milling on glassy arsenic monoselenide g-AsSe is recognized with XRPD analysis applied to the arrangement of diffuse peak-halos ascribed to intermediate- and extended-range ordering, respectively revealed in the first sharp diffraction peaks (FSDP) and second sharp diffraction peaks (SSDP). These features are interpreted within the modified microcrystalline approach, accepting that peak-halos in the XRPD patterns of amorphous substances originate from the superposition of inter-planar- and inter-atomic correlations due to quasi-crystalline remnants, the former contributing through broadened reflexes from quasi-crystalline planes (Bragg diffraction contribution), and the latter contributing through inter-atomic distances within these planes and inter-molecular correlations related to As_4_Se_4_ molecules (Ehrenfest diffraction contribution).

Mixed molecular-network elements stabilized in g-AsSe by rapid melt-quenching are destroyed under high-energy mechanical milling due to a large number of different structural defects with unfavorable energies. Milling destroys cage-like As_4_Se_4_ molecules, facilitating the transition to a polymerized preferential chain-like network. The effect of nanomilling-driven reamorphization is shown to result in the increased FSDP position and width, resulting in a fragmentation impact on the correlation length of the FSDP-responsible entities. Under nanomilling, destruction in the intermediate-range order is accompanied by changes in the extended-range order, which is revealed through a high-angular shift in the SSDP position and broadening in the SSDP width. A breakdown in the intermediate-range order is revealed in the destruction of most distant inter-atomic correlations, which belong to remnants of some quasi-crystalline planes, whereas the longer correlations dominate in the extended-range order.

The respective pathways of milling-driven reamorphization in arsenic monoselenide connected with the As_4_Se_4_ molecule and iso-compositional network-forming units are identified employing ab initio quantum-chemical cluster modeling code (CINCA).

## Figures and Tables

**Table 1:** Parameterization of diffuse peak-halos in MQ-derived (unmilled) and MM-derived (nanomilled) g-AsSe.

Sample	Peak-Halo	Peak Position, θ	Peak Width, *FWHM*	Characteristic Distance, *R*	Correlation Length, *L*	Interatomic Distance, *d_s_*
		°2θ	°2θ	Å	Å	Å
As_50_Se_50_, unmilled	Pre-FSDP	6.22	4.01	14.2	22.0	17.44
	FSDP	16.03	3.21	5.5	27.5	6.79
	Post-FSDP	19.96	4.24	4.4	20.8	5.46
	SSDP	29.77	5.88	3.0	15.0	3.68
	Post-SSDP	33.91	7.07	2.6	12.5	3.24
	TDP	52.64	8.77	1.7	10.1	2.14
As_50_Se_50_, nano-milled	Pre-FSDP	6.21	4.69	14.2	18.8	17.49
	FSDP	16.42	4.27	5.4	20.7	6.63
	Post-FSDP	20.81	3.63	4.3	24.3	5.25
	SSDP	30.88	7.02	2.9	12.6	3.56
	Post-SSDP	35.88	6.41	2.5	13.8	3.08
	TDP	52.89	8.87	1.7	10.0	2.13

## Data Availability

The data presented in this study are available on request from the corresponding author.
